# Knowledge and Utilization of Computers Among Health Professionals in a Developing Country: A Cross-Sectional Study

**DOI:** 10.2196/humanfactors.4184

**Published:** 2015-03-26

**Authors:** Kalid Alwan, Tadesse Awoke, Binyam Tilahun

**Affiliations:** ^1^Harar Health Science CollegeHararEthiopia; ^2^Department of Epidemiology and BiostatisticsUniversity of GondarGondarEthiopia; ^3^Institute of Medical InformaticsUniversity of MünsterMünsterGermany

**Keywords:** computer literacy, health professionals, eHealth success, Ethiopia

## Abstract

**Background:**

Incorporation of information communication technology in health care has gained wide acceptance in the last two decades. Developing countries are also incorporating information communication technology into the health system including the implementation of electronic medical records in major hospitals and the use of mobile health in rural community-based health interventions. However, the literature on the level of knowledge and utilization of information communication technology by health professionals in those settings is scarce for proper implementation planning.

**Objective:**

The objective of this study is to assess knowledge, computer utilization, and associated factors among health professionals in hospitals and health institutions in Ethiopia.

**Methods:**

A quantitative cross-sectional study was conducted on 554 health professionals working in 7 hospitals, 19 primary health centers, and 10 private clinics in the Harari region of Ethiopia. Data were collected using a semi-structured, self-administered, and pre-tested questionnaire. Descriptive and logistic regression techniques using SPSS version 16.0 (IBM Corporation) were applied to determine the level of knowledge and identify determinants of utilization of information communication technology.

**Results:**

Out of 554 participants, 482 (87.0%) of them responded to the questionnaire. Among them, 90 (18.7%) demonstrated good knowledge of computers while 142 (29.5%) demonstrated good utilization habits. Health professionals who work in the primary health centers were found to have lower knowledge (3.4%) and utilization (18.4%). Age (adjusted odds ratio [AOR]=3.06, 95% CI 0.57-5.37), field of study (AOR=3.08, 95% CI 1.65-5.73), level of education (AOR=2.78, 95% CI 1.43-5.40), and previous computer training participation (AOR=3.65, 95% CI 1.62-8.21) were found to be significantly associated with computer utilization habits of health professionals.

**Conclusions:**

Computer knowledge and utilization habits of health professionals, especially those who work in primary health centers, were found to be low. Providing trainings and continuous follow-up are necessary measures to increase the likelihood of the success of implemented eHealth systems in those settings.

## Introduction

### Background

The use of information communication technology in health care is not merely about technology but a means to solve the critical data management and clinical communication challenges in health care organizations, especially in developing countries [1]. Given the high burden of disease and the low number of skilled personnel, eHealth is believed to improve health care by strengthening the health system, supporting delivery of care, and improving communication among different health care organizations and professionals [2,3]. Incorporation of information communication technology in developing countries has gained wide acceptance in the last several decades with different success stories in different sectors, especially in the business sector [4]. However, when compared with other sectors, only a limited application of information technology advancements is seen in health care organizations [5,6].

Recently there has been an increase in the implementation of eHealth applications in developing countries that includes telehealth, mobile health applications, electronic medical records, and health information management systems [7]. However, most implementations remain in the pilot phase because of different technical and personnel issues [8]. Most evaluations and case studies from previous implementations in those settings report that infrastructural challenges and the existing skill levels of health professionals are the most common obstacles to the success of implemented eHealth systems [8,9,10]. However, the literature on the level of knowledge and utilization of health professionals and their current exposure in information communication technology use is scarce.

For Ethiopia, with its population of approximately 80 million people, poor health system, and severe shortage of health professionals, incorporation of eHealth to the different sectors of the system is regarded as the only way to achieve the country’s goal of universal health coverage by 2020. For that, the government is currently implementing different eHealth initiatives, and the Health Sector Development Plan IV [11] strategy is in progress to transform health services into a cost effective and efficient system. The Ministry of Health of Ethiopia is also drafting a new national eHealth strategy [12] following the recently published World Health Organization guideline [13] on eHealth strategy development. To ensure sustainability, the country is also teaching health informatics professionals [14] who support different eHealth implementation initiatives in the country.

### Statement of the Problem

With the new initiatives in Ethiopia, expanded implementation of eHealth is expected in the coming years, but these systems must be used effectively to meet objectives; this is entirely dependent on health professionals’ use of eHealth in their daily tasks. Studies in similar settings show that that lack of basic knowledge of computers and software on the part of health professionals is a main factor in failure of eHealth systems [5,15-18]. Therefore, before costly implementation, it is necessary to know the current knowledge and utilization habits of health professionals so that effective prior planning can take place. To our knowledge, there is little existing evidence in primary care and hospital contexts in developing countries. This study aims to fill this gap.

### Objectives

The goal of this study is to assess the current levels of knowledge and utilization of computers among health professionals and identify factors affecting utilization. The outcome of this research will help evidence-based planning and implementation of eHealth in Ethiopia and generate additional insight on the topic for further development of health systems in other developing countries.

##  Methods

### Overview

Institution-based quantitative cross-sectional research was conducted in 7 hospitals, 19 primary health centers, and 10 private clinics which are on the frontline to implement different eHealth applications in the coming year. All health professionals working at these health institutions were included in the study. There were 621 health professionals working at those institutions; all except those on annual and sick leave were included in the study.

A pre-tested, self-administered questionnaire, adapted from a previous study [19](Multimedia Appendix 1), was used to collect data on sociodemographic characteristics and computer knowledge and utilization by health professionals. The questionnaire was prepared in English. The data collection was facilitated by eight information technology diploma holders, and supervision was done by the principal investigator.

In this study, health professionals were defined as those employees with at least a diploma certificate in the health professions who are practicing clinical service in the study settings. Computer knowledge was defined as a basic understanding about computers and how to use them. It involves knowing hardware and software, what a computer virus is, and how to manage files and use basic computer applications like a computer network and the Internet. Twenty questions were used to assess computer knowledge. Utilization of computers is a basic skill and involves use of the computer and Internet; managing and storing files; and retrieving, analyzing, and presenting the data on hand. Fifteen questions were used to assess health worker computer utilization habits.

Both knowledge and utilization of computers among health professionals were classified after adopting a cut of value from the Nigerian study in 2004 on the same topic [20]. Those scoring 80% or above on the knowledge test were rated as having good computer knowledge; those scoring below 80% were rated as having poor computer knowledge**.** Those study subjects who scored 60% or above on the utilization test were rated as having good computer utilization, while those scoring below 60% were rated as having poor computer utilization.

Data were entered using Epi Info then exported to SPSS package version 16 (IBM Corporation) for analysis. Frequencies and cross tabulations were used for the descriptive analysis of the data. Associations between participant’s characteristics and knowledge and utilization of computer were analyzed using binary logistic regression.

### Ethics Statement

The ethical clearance committee of the University of Gondar College of Medicine and Health Sciences through the School of Public Health approved this study. Data were collected after getting permission and clearance from the ethical clearance committee of the Harari Regional Health Bureau. Written consent was obtained from each respondent on a form attached to the questionnaire.

## Results

### Sociodemographic Characteristics

There were 554 public health professionals who participated in this study. Among them, 482 (87.0%) correctly filled out and returned the questionnaire. The median age of respondents was 25 years; 52.0% (251/482) were male. The majority of participants (311/482, 65.0%) were nurses while 20.7% (100/482) were pharmacists and laboratory technicians. Most respondents (364/482, 75.5%) had received at least some kind of basic computer training in the past.

**Table 1:** Sociodemographic characteristics of respondents.

Predictor variables of respondents		n (%)
**Age, years**		
	≤25	174 (36.1)
	26-30	118 (24.5)
	31-35	71 (14.7)
	≥36	119 (24.7)
**Sex**		
	Male	251 (52.1)
	Female	231 (47.1)
**Profession**		
	Medical doctor, health officer	51 (10.6)
	Pharmacist, lab technician	100 (20.7)
	Nurse	311 (64.5)
	Other^a^	20 (4.1)
**Education**		
	BSc or Above	126 (26.1)
	Diploma	356 (73.9)
**Training**		
	Yes	364 (75.5)
	No	118 (24.5)

^a^Environmental health, dentistry, physiotherapy, and radiography

### Computer Knowledge

**Figure 1:**
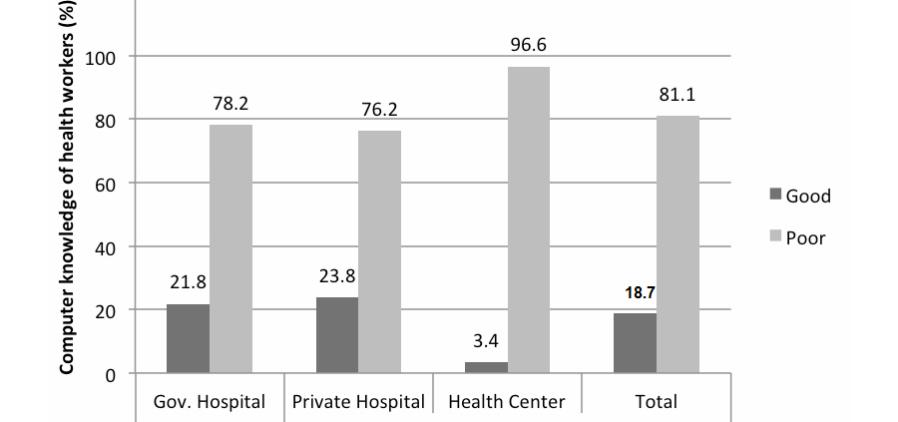
Knowledge of computers among health professionals.

Only 18.7% (90/482) of the respondents demonstrated good knowledge of computers in this study. Of them, few health professionals working at primary health centers (4/90, 4.4%) showed good computer knowledge compared to those working at government (21/90, 23.3%) and private (24/90, 26.7%) hospitals. The results are displayed in Figure 1.

### Computer Utilization

**Figure 2:**
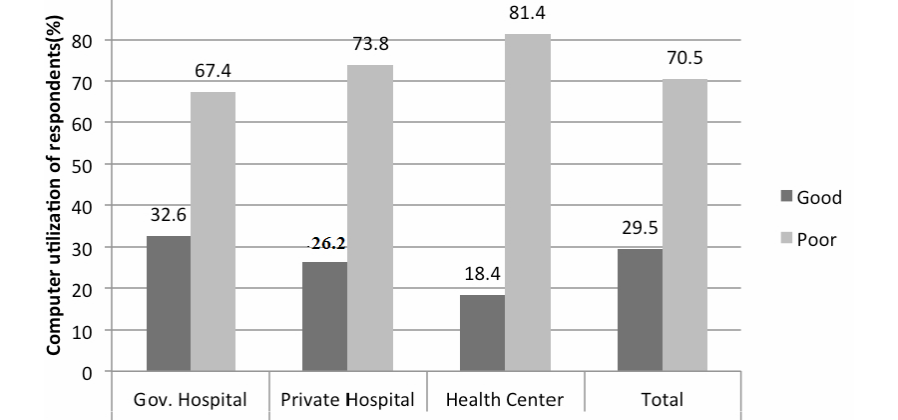
Utilization of computers among health professionals.

A total of 29.5% of the respondents (142/482) had good utilization of computers. Participants working at government hospitals showed (115/353, 32.6%) good computer utilization, which was higher than those at private hospitals (11/42, 26.2%) and much higher than those at primary health centers (16/87, 18.4%). The results are shown in Figure 2 with more detail.

### Factors Associated With Computer Utilization

With the multivariate logistic regression analysis done on computer utilization as dependent with other hypothesized independent variables, age, field of study, level of education, and computer training were found to be significantly associated with the computer utilization habits of health professionals.

To quantify each relationship, respondents who were younger (age 25-35) were approximately 3 times more likely to use computers than respondents aged 36 years and older (adjusted odds ratio [AOR]=3.06, 95% CI 0.57-5.37). Additionally, respondents who had previous computer training were 3.65 times more likely to use computers than those who did not have any kind of computer training (AOR=3.65, 95% CI 1.62-8.21). In the professional category, medical laboratory technicians and pharmacists were more likely to use computers than nurses (AOR=3.08, 95% CI 1.65-5.73). Additionally, those with higher levels of education were 2.78 times more likely to use computers than those with lower levels of education (AOR=2.78, 95% CI 1.43-5.40). The results are shown in Table 2.

**Table 2:** Factors associated with computer utilization among health professionals.

Predictor variables		Utilization		COR^a^ (95% CI)	AOR (95% CI)
		Good	Poor		
**Age**					
	≤25	54	120	2.36 (1.31-4.25)	1.16 (0.40-3.34)
	26-35	46	72	5.36 (1.81-6.21)	3.06 (0.57-5.37)^c^
	≥36	19	100	1	1
**Sex**					
	Male	94	157	2.28 (1.51-3.43)	1.05 (1.01-2.69)
	Female	48	183	1	1
**Marital status**					
	Never married	75	127	1.87 (1.26-2.79)	1.40 (0.79-2.69)
	Married	67	213	1	1
**Profession**					
	Medical doctor, health officer	28	23	4.51 (2.44-8.35)	1.89 (0.77-4.61)
	Pharmacist, lab technician	39	61	2.37 (1.46-3.85)	3.08 (1.65-5.73)^c^
	Nurse	66	245	1	1
	Other^b^	9	11	3.03 (1.26-7.63)	1.30 (0.70-7.50)
**Education**					
	BSc or above	66	60	4.05 (2.63-6.24)	2.78 (1.43-5.40)^c^
	Diploma	76	280	1	1
**Position**					
	Institution head	6	4	3.93 (1.08-14.20)	1.97 (0.34-11.24)
	Team leader	28	53	1.38 (0.83-2.30)	1.70 (0.85-3.38)
	Care provider	108	283	1	1
**Training**					
	Yes	133	231	6.97 (3.42-14.21)	3.65 (1.62-8.21)^c^
	No	9	109	1	1
**Service year**					
	6-10	32	49	3.02 (3.27-24.77)	1.51 (1.92-22.02)
	11-15	9	45	2.76 (0.86-8.76)	1.28 (0.62-8.40)
	≥16	5	69	1	1

^a^Crude odds ratio

^b^Environmental health, dentistry, physiotherapy, and radiography

^c^Significant at *P*<.05.

## Discussion

### Principal Findings

The findings of this study show that computer knowledge and utilization was generally low and was lower for public health professionals who work in the primary health care centers. The results are lower compared to findings in previous studies [2,20,21], which might be attributed to a difference in study participants between the studies; previous studies only included health professionals working in hospitals while this study also includes health professionals working at the health centers, which have less computer access and information communication technology infrastructure.

The analysis of the determinant factors of computer utilization shows that age, field of study, level of education, and computer training were found to be significantly associated with computer utilization. Among the factors, stronger association was found with computer training. The result is consistent with previous studies [20-23]. This result implies that providing trainings—not only about the specific eHealth software application which is going to be implemented but also generally about computers—can make a significance difference in system adaptation in health care.

In this study, we found that younger health professionals are more likely to use eHealth systems than older health professionals, which is consistent with other studies [24-27]. This implies that older employees need more assistance to adapt to and use the system.

Additionally, health professionals with advanced levels of education showed significantly better computer utilization than middle-level health professionals. The result is not surprising given the increasing number of computer-based tasks associated with further studies. Finding of this study was inconsistent with studies in India which showed that level of education was not significantly associated with computer utilization [21,22]. This may be due to training differences for health professionals in India and Ethiopia in which the basic computer courses in Ethiopia are more theoretical with few hours of practical lessons.

As skill is a main factor in eHealth success [28], interventions are needed to increase health professionals’ knowledge and utilization. The Ministry of Health should provide training to the health professionals so that their knowledge can increase and their anxiety about technology can decrease. In addition, it is necessary to increase accessibility to computers, especially in primary care health centers, so health professionals can practice using computers in different activities before the main eHealth system is implemented.

In this study, knowledge and utilization habit measurements were self-reported, which might have some response bias. A further study complemented by qualitative approach is recommended to give more insight on how actual computer knowledge and utilization habits contribute to a better adoption of eHealth systems.

### Limitations of the Study

This study did not address the attitude of health workers towards computers, which can influence their computer knowledge and utilization. Additionally, the information collected was self-perceived, which might have reporting bias. Future studies including attitude and actual practical use assessment are recommended. Additionally, the relationship between computer knowledge and use on eHealth success needs further investigation.

###  Conclusions

Computer knowledge and utilization habits of health professionals, especially those who work in primary health centers, were found to be low. Providing trainings and continuous follow-up are necessary measures to increase the likelihood of the success of implemented eHealth systems in those settings.

## Multimedia Appendix 1

Questionaire.

## Abbreviations

COR: crude odds ratio
AOR: adjusted odds ratio
