# Supplementary material for: Knowledge and Utilization of Computers Among Health Professionals in a Developing Country: A Cross-Sectional Study
Source: JMIR Hum Factors. 2015 Mar 26;2(1):e4. doi: 10.2196/humanfactors.4184 (PMC4797659; doi:10.2196/humanfactors.4184)
Supplement: Supplementary file 1 [file humanfactors_v2i1e4_app1.pdf]

<u>Quantitative data Instrument</u>

A. Informed consent statement

Dear Sir/Madam,

The purpose of this Questionnaire is to collect information on knowledge and utilization of computers among health professionals to use it as an input for its future improvement. **You are chosen to participate in this study by chance and your anonymous answers will be used only for research purpose.** The results of the study will benefit the the management and the hospital by producing relevant information on the current level of knowledge and utilization habit of the staff.

In order to effectively attain the purpose of the research I request you to give **<u>genuine</u>** response to each question. There are questions for you to complete and there is no need to put your name on the questionnaire; no individual responses will be reported. Your answers are completely confidential. It is your full right to refuse, to answer any or all of the questions. If you don't want to participate you can leave the questionnaire empty. However, your honest response to these questions will help us achive the objective of this study.

B. **Certificate of consent**

I understand that the findings of this research will be disseminated to Hospital management and decision makers that will be useful as an input for intervention design.

I voluntarily consent to participate in this study.

I agree ☐ I disagree ☐

If you are agreeable to participate in the study please visit the next page.

For any further question, contact the investigator

Thank you for consenting to be a participant in this study.

For Data collectors use only:
Institution:________________________ Questionnaire ID: ___________

B. SOCIO DEMOGRAPHIC DATA

S.No	QUESTIONS	CODING
201	Age in year	________
202	Sex	1. Male<br>2. female
203	Marital status	1. Sing<br>2. Married<br>3. Divorced<br>4. Widowed<br>5. Separated
204	Religion	1. Muslim<br>2. Orthodox<br>3. Protestant<br>4. Catholic<br>5.Other specify__________
205	monthly income in Ethiopian Birr	__________
206	Job satisfaction index	1. Very strongly satisfied<br>2. Very satisfied<br>3. Satisfied<br>4. Poorly satisfied<br>5. Unsatisfied
207	Profession	1. Medical Doctor<br>2. Nurse<br>3. Health Officer<br>4. Lab. Tech.<br>5. Phar. Tech<br>6. Environmental Health<br>7.Other:specify____________
208	Educational status	1. Sub specialist<br>2. Specialist<br>3. MSc<br>4. BSc<br>5. Diploma
209	At which department do you work?	1.Out patient department<br>2. inpatient department

		3.emergency department<br>4.pharmacy department<br>5.labratory department<br>6.other:specify______________
2010	What is your work position?	1.Health care provider<br>2.department/team leader<br>3.head of institution<br>4.other:specify_____________
2011	How many service year(s) do you have?	_____________
2012	Do you have any kind of computer training?	1. Yes<br>2. No
2013	If yes for the above question what type of computer training do you have?	1.short term on job training from office<br>2.short term training at training center<br>3.formal training at university or college<br>4.personal effort/informal training<br>5.others:<br>specify________________
2014	Do you have access/ the opportunity/ to use computer?	1. Yes<br>2. No
2015	If yes for the above question where do you get it?	1.at office<br>2.tele centers<br>3.private computer centers<br>4.i have computer so use it<br>5.from friends/colloquies<br>6.other place: specify___<br>__________________

C. KNOWLEDGE OF COMPUTER

S.N	QUESTION	CODING
301	The physical part of a computer system is __	1. Software<br>2. Hardware<br>3. I don’t know
302	Which of the following is an example of hardware?	1. Ms Office<br>2. Central processing unit CPU)<br>3. Windows XP<br>4. I don’t know
303	____ is programs or instructions that controls coordinate and manage the activities of computer system.	1. Software<br>2. Hardware<br>3. I don’t know
304	Application and system software are the two main type of computer software?	1. Yes<br>2. No<br>3. I don’t know
305	Application software is an operating systems which control the workings of the computer	1. Yes<br>2. No<br>3. I don’t know
306	Communication between personal computers made by different manufacturers is impossible?	1. Yes<br>2. No<br>3. I don’t know
307	Digital sounds like audio and video data cannot be communicated over a network?	1. Yes<br>2. No<br>3. I don’t know
308	A computer network is an interconnected of computers located in different places.	1. Yes<br>2. No<br>3. I don’t know
309	Computer networks do not allow different personal computers to access the same files.	1. Yes<br>2. No<br>3. I don’t know
3010	Internet is a global communication of network	1. Yes<br>2. No<br>3. I don’t know
3011	Companies that use e-mail no longer need to use the postal system	1. Yes<br>2. No<br>3. I am not sure

3012	What is WWW stands for?	______________________
3013	Which storage device has a larger capacity?	1. Floppy disc<br>2. CD<br>3. DVD<br>4. Flash disk with 2 gb
3014	The speed of a computer is measured by its_____	1.Random-access Memory (RAM)<br>2. Moniter<br>3.other: specify__________<br>4. I don't know
3015	Modem allows computers to communicate using telephone line.	1. Yes<br>2. No<br>3. I don't know
3016	People who work at home never communicate with their office using the network.	1. Yes<br>2. No<br>3. I am not sure
3017	Which one of the following is Information Highway (communication media?)	1. Phone lines<br>2. Satellites<br>3. Fiber-optic cables<br>4. All<br>5. I don't know
3018	Computer virus is____________	1. software<br>2. hardware<br>3.I don't know
3019	Computers only understand the series of 1's and 0's?	1. Yes<br>2. No<br>3. I don't know
3020	Which software is used for analysis of statistical data?	1. Epi Info<br>2. SPSS<br>3. both<br>4.others:specify________________<br>5. I don't know

D. Utilization of Computers

S.N	QUESTION	CODING
401	Which search engines do you use?	1. Google<br>2. Yahoo<br>3. Both<br>4. None of them
402	Do you have an e-mail address?	1. Yes<br>2. No
403	Do you have access to medical journal/ organization subscription online?	1. Yes<br>2. No
404	For what purpose do you use the Internet?	1. E-Mail<br>2. Research<br>3. Film/News<br>4. Scholarship<br>5.Others purpose: specify__________<br>6. I don't use internet
405	How often do you access the Internet in a month?	____________________
406	which of the following symbols used to save a file using key board	1. Ctrl + S<br>2. Ctrl + C<br>3. Ctrl + X<br>4. I don't know
407	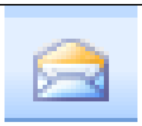 this symbol represents for ______	1. E-mail toolbar button<br>2. Save toolbar button<br>3. Pest toolbar button<br>4. Cut toolbar button<br>5. I don't know
408	How many hours you spend on the computer per week?	__________________
409	Have you ever store your data in computer?	1. Yes<br>2. No
4010	If yes which type of storage device do you use?	1. Compact disk (CD)<br>2. Digital Versatile Disk (DVD)

		3. Flash disk<br>4. Hard disk<br>5. Other: specify_________
4011	How many times do you use MS-office for writing report in a year?	_______________
4012	Have you ever used statistical packages to analyze your data?	1. Yes<br>2. No
4013	If yes which type of statistical package do you used?	1. SPSS<br>2. Epi Info<br>3. STATA<br>4.Other:specify___________
4014	Have you ever used mail for reporting?	1. Yes<br>2. No
4015	If yes which method do you prefer?	1. Postal<br>2. E-mail<br>3. Fax<br>4.Other:specify___________

Thank you for participating in this study!
